# Soluble Angiotensin-Converting Enzyme 2 Protein Improves Survival and Lowers Viral Titers in Lethal Mouse Model of Severe Acute Respiratory Syndrome Coronavirus Type 2 Infection with the Delta Variant

**DOI:** 10.3390/cells13030203

**Published:** 2024-01-23

**Authors:** Cosimo Cianfarini, Luise Hassler, Jan Wysocki, Abdelsabour Hassan, Vlad Nicolaescu, Derek Elli, Haley Gula, Amany M. Ibrahim, Glenn Randall, Jack Henkin, Daniel Batlle

**Affiliations:** 1 Division of Nephrology/Hypertension, Department of Medicine, Feinberg School of Medicine, Northwestern University, 710 North Fairbanks Court, Chicago, IL 60611, USA; 2 Charité Universitätsmedizin Berlin, 10117 Berlin, Germany; 3 Howard Taylor Ricketts Laboratory, Department of Microbiology, The University of Chicago, Lemont, IL 60637, USA; 4 Center for Developmental Therapeutics, Northwestern University, Evanston, IL 60208, USA

**Keywords:** SARS-CoV-2, COVID-19, angiotensin-converting enzyme 2, ACE2

## Abstract

Severe acute respiratory syndrome coronavirus type 2 (SARS-CoV-2) utilizes angiotensin-converting enzyme 2 (ACE2) as its main receptor for cell entry. We bioengineered a soluble ACE2 protein termed ACE2 618-DDC-ABD that has increased binding to SARS-CoV-2 and prolonged duration of action. Here, we investigated the protective effect of this protein when administered intranasally to k18-hACE2 mice infected with the aggressive SARS-CoV-2 Delta variant. k18-hACE2 mice were infected with the SARS-CoV-2 Delta variant by inoculation of a lethal dose (2 × 10^4^ PFU). ACE2 618-DDC-ABD (10 mg/kg) or PBS was administered intranasally six hours prior and 24 and 48 h post-viral inoculation. All animals in the PBS control group succumbed to the disease on day seven post-infection (0% survival), whereas, in contrast, there was only one casualty in the group that received ACE2 618-DDC-ABD (90% survival). Mice in the ACE2 618-DDC-ABD group had minimal disease as assessed using a clinical score and stable weight, and both brain and lung viral titers were markedly reduced. These findings demonstrate the efficacy of a bioengineered soluble ACE2 decoy with an extended duration of action in protecting against the aggressive Delta SARS-CoV-2 variant. Together with previous work, these findings underline the universal protective potential against current and future emerging SARS-CoV-2 variants.

## 1. Introduction

Since its first appearance in Wuhan, China, in December 2019, severe acute respiratory syndrome coronavirus type 2 (SARS-CoV-2) has spread worldwide, posing a threat to public health [1,2]. Four years later, and despite massive improvements largely because of the vaccination efforts, new cases are frequently reported [3,4]. Omicron variants are currently the most common variants seen worldwide [5,6,7]. SARS-CoV-2, like other coronaviruses, including its predecessor SARS-CoV-1, uses membrane-bound angiotensin-converting enzyme 2 (ACE2) as its primary cell entry receptor [8,9,10,11]. The complex multistep process of SARS-CoV-2 cell entry starts with the interaction of the receptor-binding domain (RBD) of the viral S1 spike protein with ACE2 on the cell surface [12,13,14,15]. Conformational changes then lead to priming of the viral spike protein by proteases like transmembrane protease serine 2 (TMPRSS2), which results in fusion pore formation and, ultimately, cell infection [9,16,17,18].

At the beginning of the COVID-19 pandemic in early 2020, our group proposed the use of soluble ACE2 proteins to intercept the binding of SARS-CoV-2 to its receptor, the membrane-bound ACE2, via the so-called “decoy effect” [19,20]. We bioengineered a human soluble ACE2 protein that was shortened from 740 to 618 amino acids and fused with an albumin-binding domain (ABD) to increase its duration of action [21]. Later, a dodecapeptide (DDC) motif that contained four cysteine residues was introduced to enhance binding affinity for SARS-CoV-2 via the formation of a disulfide-bonded dimeric protein [21,22]. In human kidney organoids and the k18-hACE2 mouse model of lethal SARS-CoV-2 infection with the ancestral virus variant, we demonstrated the potential of our bioengineered soluble ACE2 proteins to neutralize SARS-CoV-2, markedly improve survival and provide in vivo lung, brain and kidney protection [21,22]. We established preventative, intranasal delivery, starting one hour before viral inoculation, followed by two additional doses at 24 and 48 h post-viral inoculation, as an effective mode of ACE2 618-DDC-ABD delivery, achieving optimal protection in terms of survival and organ injury in the k18-hACE2 mouse model infected with ancestral SARS-CoV-2 [23].

As new variants of SARS-CoV-2 emerge, mutational escape and resistance to treatment are critical issues, and both antibodies and antiviral drugs have been shown to be associated with the development of the mutational escape of SARS-CoV-2 [24,25]. Mutations of SARS-CoV-2 that would decrease ACE2 decoy affinity while simultaneously maintaining the efficacious binding of SARS-CoV-2 to the membrane-bound ACE2 receptor have not been described and are unlikely to occur [26,27]. This highlights an advantage of soluble ACE2 proteins over monoclonal antibodies [28]. Moreover, studies in permissive cells have shown that soluble ACE2 proteins are effective against different SARS-CoV-2 variants [10,21,22,23,29,30,31]. The aim of this study is to demonstrate the efficacy of our unique soluble ACE2 protein against the otherwise lethal infection caused by the SARS-CoV-2 Delta variant, which causes severe disease in humans. Moreover, ACE2 618-DDC-ABD was administered as early as six hours prior to viral inoculation to further determine its lasting protective effect and demonstrate its extended duration of action when applying the protein preventatively before infection.

## 2. Materials and Methods

### 2.1. In Vivo Infectivity Studies

All in vivo infectivity studies were performed at the BSL-3 facility of the Ricketts Regional Biocontainment Laboratory. k18-hACE2 mice were purchased from The Jackson Laboratory (Bar Harbor, ME, USA) at 6–9 weeks of age. k18-hACE2 mice express full-length human ACE2 (hACE2) with an estimated hACE2 gene copy number of 8 [32]. This number was found to provide optimal susceptibility to SARS-CoV infection by McCray et al. [32]. To our knowledge, sex differences in the gene copy number have not been described. k18hACE2 mice are also susceptible to SARS-CoV-2, and infection with a high viral load is lethal [22,23,33,34,35,36,37,38]. As most SARS-CoV-2 variants bind mouse ACE2 poorly, the transgenic expression of hACE2 is necessary to allow the virus to establish a virulent infection [39].

After acclimatation to the BSL-3 facility for at least a week, animals were infected by highly trained personnel using intranasal inoculation of 2 × 10^4^ plaque-forming units (PFUs) of SARS-CoV-2 Delta variant (Isolate hCoV-19/USA/MD-HP05647/2021 Lineage B.1.617.2; Delta variant) in a volume of 20 μL per animal under light anesthesia. Disease severity in k18-hACE2 mice infected with the SARS-CoV-2 Delta variant has been reported to be similar to or greater than infection with the ancestral virus, where animals typically succumb to disease by days 5–9 post-viral inoculation [22,23,33,34,35,36,40,41].

### 2.2. Administration of ACE2 618-DDC-ABD

For the following experiments, two groups of k18-hACE2 mice (n = 10 per group, 5 male and 5 female each) were administered with either ACE2 618-DDC-ABD or phosphate-buffered saline (PBS) as vehicle controls were investigated. The number of animals per experimental group was deemed sufficient based on previously performed studies of SARS-CoV-2-infected k18-hACE2 mice by our group [22,23] and others [42,43]. ACE2-618-DDC-ABD was administered intranasally (10 mg/kg BW in a volume of 30 μL per animal) for a total of three doses. The first dose was given six hours before inoculation of the animal with the SARS-CoV-2 Delta variant, followed by the same dose 24 and 48 h after viral inoculation. PBS as vehicle control was administered following the same protocol at the same time points.

### 2.3. Evaluation of Body Weight and Clinical Score

Following inoculation of the animals with the SARS-CoV-2 Delta variant, all mice were monitored for health two to three times a day using a clinical scoring system that takes into account behavior and appearance of the animals. Details on this clinical score have been previously reported by our group [22,23]. Additionally, animals were weighed once daily throughout the study period. Mice that lost more than 20% of their initial body weight and did not regain it within two days or had a severely worsened clinical score were humanely euthanized as per study protocol. This was considered a fatal event.

To compare viral titers at similar time points, randomly selected animals of each sex from both groups were euthanized on day six post-viral inoculation, together with the animals that were euthanized because of disease severity. All remaining mice were monitored for up to 14 days at the BSL-3 facility of the Ricketts Regional Biocontainment Laboratory and euthanized at day 14 at the end of the study protocol if not previously deceased. Mice were euthanized using CO_2_-forced inhalation. After the last breathing movement, cervical dislocation was performed, and lung, brain and kidney tissue were collected for plaque assay analysis (see below).

### 2.4. Plaque Assay

Lung, brain and kidney tissue was removed from all euthanized animals and used for viral load measurements using plaque assay. Organs from one mouse in the PBS control group could not be collected, and the animal was, therefore, not included in the plaque assay analysis.

Tissue samples for plaque assay analysis were collected in Dulbecco’s Modified Eagle Medium (DMEM) with 2% fetal bovine serum (FBS). Samples were then homogenized and centrifuged at 1000× *g* for five minutes. The supernatant was serially diluted 10-fold and used to infect Vero E6 cells for one hour. Inoculum was removed, and 1.25% methylcellulose DMEM solution was added to the cells, followed by incubation for three days. Plates were then fixed in 1:10 formalin for one hour, stained with crystal violet for one hour and counted to determine PFUs expressed as PFU/mL.

### 2.5. Statistical Analysis

Statistical analysis and figure preparation were performed using GraphPad Prism v. 9.5.1 (GraphPad Software, Boston, MA, USA). Normal distribution was tested using the Shapiro–Wilk test. When data were normally distributed, differences between two groups were analyzed using unpaired *t*-test. Differences between groups with non-normally distributed data were analyzed using Mann–Whitney test. Differences in survival were analyzed using log-rank (Mantel–Cox) test. A *p*-value < 0.05 was considered significant.

## 3. Results

### 3.1. Survival of k18-hACE2 Mice Infected with SARS-CoV-2 Delta Variant

**Figure 1:**
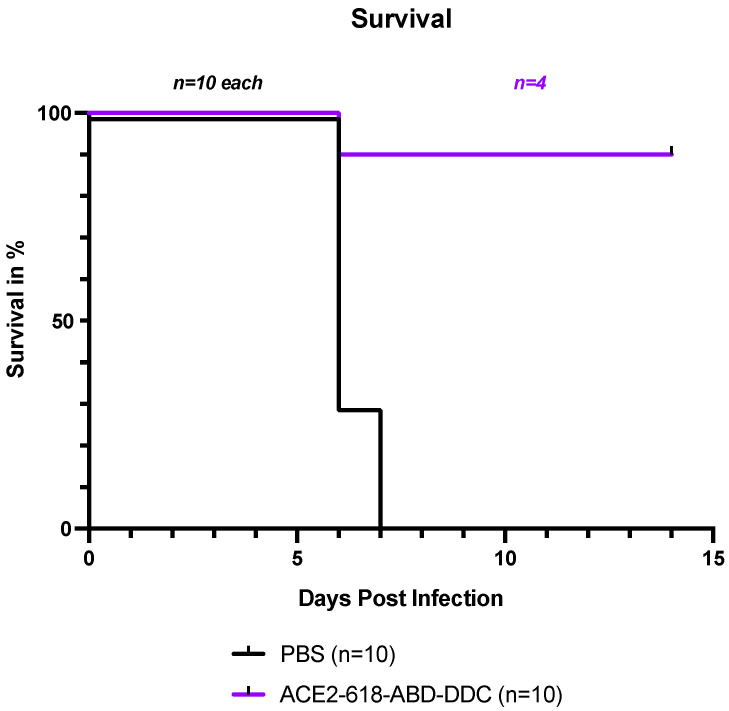
Survival of k18-hACE2 mice infected with the SARS-CoV-2 Delta variant that received either ACE2 618-DDC-ABD or PBS. Infected animals in the PBS control group (black) showed uniform lethality, and survival by day seven post-viral inoculation was 0%. In contrast, in the ACE2 618-DDC-ABD-treated group (purple), survival was 90% by day six post-viral inoculation, with only one mouse having to be humanely euthanized on day six. Five mice from the ACE2 618-DDC-ABD-treated group that were healthy according to their clinical score and weight were sacrificed on day six post-viral inoculation for comparison of viral titers at similar time points. The remaining four mice in the ACE2-618-DDC-ABD-treated group all survived until the end of the study protocol at day 14 post-viral inoculation. The difference in survival was statistically significant (*p* = 0.0004 using log-rank (Mantel–Cox) test).

All infected k18-hACE2 mice that received PBS either died or had to be humanely euthanized by day seven post-viral inoculation as per the study protocol (Figure 1, black). In contrast, the survival of the mice that received ACE2 618-DDC-ABD was 90% on day six post-viral inoculation. Only one mouse in this group had to be humanely euthanized on day six post-viral inoculation (Figure 1, purple). To compare viral titers at similar time points, five healthy mice from the ACE2-618-DDC-ABD-treated group were sacrificed on day six post-viral inoculation. The remaining four mice in this group all survived until day 14, the end of the study period. This marked difference in survival was statistically significant (*p* = 0.0004 using log-rank (Mantel–Cox) test).

### 3.2. Clinical Score of k18-hACE2 Mice Infected with SARS-CoV-2 Delta Variant

**Figure 2:**
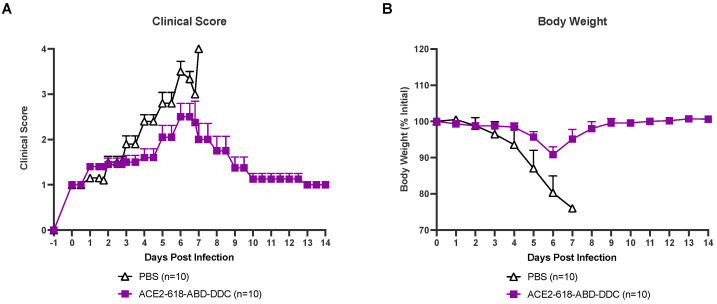
Clinical score and body weight of k18-hACE2 mice infected with the SARS-CoV-2 Delta variant that received either ACE2 618-DDC-ABD or PBS. (Panel (**A**)) Infected animals in the PBS control group (white) developed severe disease, and their clinical scores increased accordingly until days six and seven post-viral inoculation by which time point, all mice had either died or had to be humanely euthanized. In contrast, animals in the ACE2 618-DDC-ABD-treated group (purple) developed less severe disease, and their clinical scores were lower compared to the PBS control group. All remaining animals from the ACE2 618-DDC-ABD-treated group had recovered by the end of the 14-day study period. (Panel (**B**)) Infected animals in the PBS control group (white) lost up to ~20% of their initial body weight until days six and seven post-viral inoculation by which time point, all mice had either died or had to be humanely euthanized. In contrast, animals in the ACE2 618-DDC-ABD-treated group (purple) lost, on average, less than ~10% of their initial body weight by days six and seven post-viral inoculation. All remaining animals from the ACE2 618-DDC-ABD-treated group recovered stable body weight by the end of the 14-day study period.

All infected animals were evaluated for health using a clinical score two to three times a day (see Material and Methods section). Infected k18-hACE2 mice that received PBS as the vehicle control developed severe disease, and their clinical scores increased until day six and seven post-viral inoculation (Figure 2A, white). At that point, all animals in the PBS vehicle group had either died or had to be humanely euthanized as per the study protocol (Figure 1). Animals that received ACE2 618-DDC-ABD developed less severe disease, and their clinical scores were lower (Figure 2A, purple). By the end of the 14-day study period, all remaining mice in the ACE2 618-DDC-ABD group had recovered.

### 3.3. Weight Loss of k18-hACE2 Mice Infected with SARS-CoV-2 Delta Variant

All infected mice were weighed once daily throughout the 14-day study period. Infected mice that received PBS lost up to ~20% of their initial body weight until days six and seven post-viral inoculation. At this point, all mice from the PBS control group either died or had to be humanly euthanized (Figure 2B, white). Mice that received ACE2 618-DDC-ABD lost, on average, less than ~10% of their initial body weight by day six post-viral inoculation, and the remaining mice in this group recovered stable body weight toward the end of the 14-day study period (Figure 2B, purple).

### 3.4. Lung Virial Titers of k18-hACE2 Mice Infected with SARS-CoV-2 Delta Variant

**Figure 3:**
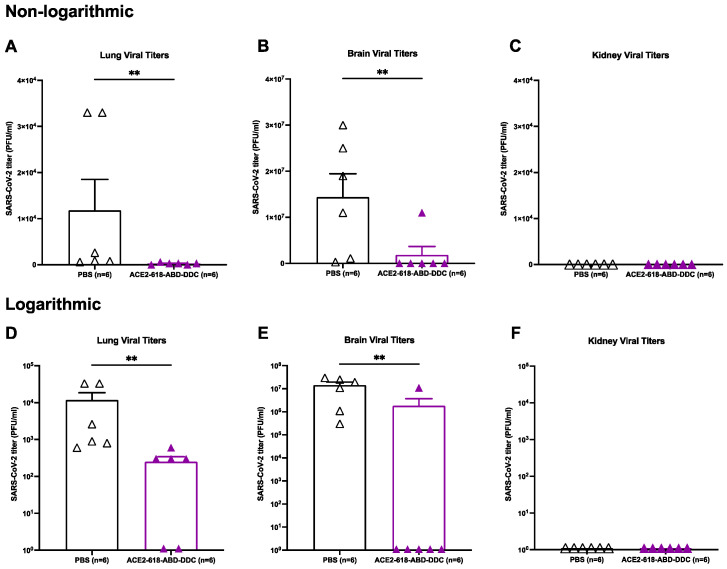
Lung, brain and kidney SARS-CoV-2 titers of k18-hACE2 mice infected with the SARS-CoV-2 Delta variant that received either ACE2 618-DDC-ABD or PBS. Organs were collected on day six post-viral inoculation. The data in the upper panels (Panels (**A**–**C**)) are also shown on a logarithmic scale in the lower panels (Panels (**D**–**F**)). Lung viral titers (Panel (**A**)) in infected animals from the ACE2 618-DDC-ABD-treated group (purple) were markedly reduced compared to lung viral titers in infected animals that received PBS (white) (2.5 × 10^2^ ± 0.9 × 10^2^ vs. 1.18 × 10^4^ ± 6.7 × 10^3^ PFU/mL, *p* = 0.004). Brain viral titers (Panel (**B**)) were also markedly reduced in mice that received ACE2 618-DDC-ABD (purple) compared to brain viral titers in the PBS control group (white) (1.8 × 10^6^ ± 1.8 × 10^6^ vs. 1.4 × 10^7^ ± 5.0 × 10^6^ PFU/mL, *p* = 0.008). Kidney viral titers (Panel (**C**)) could not be detected in any of the animals from either the ACE2 618-DDC-ABD-treated (purple) group or the PBS control group (white). In lower panels, the same differences are shown, but the logarithmic scale allows the visualization of some viral titers that were still detectable in lungs (Panel (**D**)) and brains (Panel (**E**)) of a few treated animals. In the kidneys (Panel (**F**)), no viral titers could be detected. ∗∗ *p* < 0.01.

Lung viral titers were assessed in all ten mice that received ACE2 618-DDC-ABD and in nine mice that received PBS. When comparing lung viral titers from animals sacrificed on day six post-viral inoculation (n = 6 per group), the lung viral titers were significantly reduced in animals that received ACE2 618-DDC-ABD compared to infected mice in the PBS control group (2.5 × 10^2^ ± 0.9 × 10^2^ vs. 1.18 × 10^4^ ± 6.7 × 10^3^ PFU/mL, *p* = 0.004) (Figure 3A). The data were also expressed logarithmically (Figure 3, lower panels) to visualize that some viral titers were still detectable at low levels in the lungs of a few treated animals (Figure 3D). When considering viral titers from lungs collected at all time points, a marked reduction in the lung viral titers in animals that received ACE2 618-DDC-ABD was seen as well (1.5 × 10^2^ ± 0.7 × 10^2^ vs. 7.8 × 10^3^ ± 4.7 × 10^3^ PFU/mL, *p* = 0.04). Lung viral titers were completely undetectable in the four remaining animals of the ACE2 618-DDC-ABD-treated group at the end of the 14-day study period.

### 3.5. Brain Viral Titers of k18-hACE2 Mice Infected with SARS-CoV-2 Delta Variant

Brain viral titers were assessed in all ten mice that received ACE2 618-DDC-ABD and nine mice that received PBS. When comparing brain viral titers in animals sacrificed on day six post-viral inoculation (n = 6 per group), the infected animals in the PBS control group had very high brain viral titers, whereas brain viral titers were significantly reduced in infected mice that received ACE2 618-DDC-ABD (1.8 × 10^6^ ± 1.8 × 10^6^ vs. 1.4 × 10^7^ ± 5.0 × 10^6^ PFU/mL, *p* = 0.008) (Figure 3B). The data were also expressed logarithmically (Figure 3, lower panels) to visualize if viral titers were detectable, which was the case in only one of the treated animals (Figure 3E). When considering viral titers from brains collected at all time points, a marked reduction in the brain viral titers in animals that received ACE2 618-DDC-ABD was seen as well (1.1 × 10^6^ ± 1.1 × 10^6^ vs. 1.2 × 10^7^ ± 3.7 × 10^6^ PFU/mL, *p* = 0.0007). Similar to the data for the lung viral titers, the brain viral titers were completely undetectable in the four remaining animals of the ACE2 618-DDC-ABD-treated group at the end of the 14-day study period.

Of note, when directly comparing viral titers in brains to lungs from the PBS control group, brain viral titers were about 1000-fold higher than lung viral titers (1.2 × 10^7^ ± 3.7 × 10^6^ vs. 7.8 × 10^3^ ± 4.7 × 10^3^ PFU/mL, *p* = 0.004). In fact, several of the infected, untreated mice had only slightly increased lung viral titers (see Figure 3A,D).

### 3.6. Kidney Viral Titers of k18-hACE2 Mice Infected with SARS-CoV-2 Delta Variant

Kidney viral titers were assessed in all ten mice that received ACE2 618-DDC-ABD and in nine mice that received PBS. No kidney viral titers could be detected in either the PBS control or the ACE2 618-DDC-ABD-treated group (Figure 3C,F). The limit of detection for the plaque assay is 10^2^ PFU/mL.

## 4. Discussion

This study demonstrates the dramatic protective effect of the soluble ACE2 decoy protein termed ACE2 618-DDC-ABD in the lethal k18-hACE2 mouse model of SARS-CoV-2 infection caused by the SARS-CoV-2 Delta variant. Animals that received ACE2 618-DDC-ABD six hours prior to, as well as 24 and 48 h after, viral inoculation had 90% survival compared to uniform lethality in the PBS control group. Disease severity, as evaluated using a clinical score and weight loss, was markedly improved in the ACE2-618-DDC-ABD-treated group. Moreover, lung and brain viral titers were markedly reduced in animals receiving ACE2 618-DDC-ABD compared to the PBS control group. At the end of the study period, on day 14 post-viral inoculation, all remaining animals in the ACE2 618-DDC-ABD-treated group had no detectable lung and brain viral titers. Taken together with our previous studies in the same mouse model infected with the ancestral SARS-CoV-2 variant (Washington isolate) [22,23], our data demonstrate remarkable efficacy of ACE2 618-DDC-ABD in preventing mortality and organ infectivity in the aggressive Delta variant. Furthermore, we also show that the intranasal administration of ACE2 618-DDC-ABD prior to viral inoculation results in a protective effect when administered at least six hours prior to viral inoculation, which expands on our previous work when ACE2 618-DDC-ABD was administered close to viral inoculation with the ancestral SARS-CoV-2 variant (one hour before).

The SARS-CoV-2 Delta variant first emerged in October 2020 and was shown to cause severe disease in humans [44,45,46,47]. Thirteen amino acid mutations have been found in the viral spike protein of the SARS-CoV-2 Delta variant compared to the ancestral virus genome [40]. Of particular interest are the mutations L452R, T478K, P681R and D614G, which have been reported to promote infectivity and immune escape by increasing the stability of the spike protein and RBD-ACE2 interaction, as well as facilitating cleavage of the spike precursor protein and promoting viral replication and transmission [48,49,50,51,52,53,54,55,56]. The infection of k18-hACE2 mice with the SARS-CoV-2 Delta variant induces distinct pathogenic patterns of respiratory disease [40,41]. Compared to infection with the SARS-CoV-2 Alpha variant, significantly more inflammation, increased production of antiviral cytokines and a greater number of activated immunological pathways have been demonstrated, which underlines the uniqueness of the host response against different SARS-CoV-2 variants [41]. k18-hACE2 mice have been widely used as a model of SARS-CoV-2 infection, showing dose-depended respiratory manifestations and lethality [22,23,57]. Lung viral titers in SARS-CoV-2-infected k18-hACE2 mice have been described to peak between two and four days post-infection [34]. Lung pathology starts early, becomes mild-to-moderate by day four and progresses to moderate-to-severe by day six post-infection [34]. Brain viral titers and pathology are typically undetectable at the early stages but progress rapidly within four to five days post-infection until mice succumb to infection [23,34]. Of note, we found that in k18-hACE2 mice infected with the SARS-CoV-2 Delta variant, brain viral titers were markedly higher than lung viral titers (Figure 3). This is in agreement with our findings in the same model infected with the ancestral virus variant and strongly suggests an important implication of brain viral invasion in causing high lethality [23].

We have previously reported that ACE2 618-DDC-ABD administered to k18-hACE2 mice both intranasally and systemically one hour before as well as 24 and 48 h after viral inoculation with the wild-type SARS-CoV-2 variant markedly improved survival and mitigated disease severity as assessed using a clinical score and weight [22]. ACE2 618-DDC-ABD also provided in vivo lung and kidney protection and reduced lung and brain viral titers [22]. As recently demonstrated by Hassler et al., the best protective effect can be achieved when administering ACE2 618-DDC-ABD intranasally to k18-hACE2 mice both before and after viral inoculation, which is superior to systemic administration at the same timepoints [23]. The administration of ACE2-618-DDC-ABD post-viral inoculation provided only partial protection compared to the untreated control group and was markedly less efficacious than the administration of ACE2-618-DDC-ABD both before and after viral inoculation [23]. Specifically, the intranasal administration of ACE2 618-DDC-ABD before and after viral inoculation resulted in 90% survival on day 6 post-viral inoculation, brain viral titers were undetectable in all mice, and lung and brain histopathology were essentially normal [23]. In the present study that followed the same protocol of the intranasal ACE2 618-DDC-ABD administration, brain viral titers were similarly undetectable in the ACE2 618-DDC-ABD-treated group except for one animal that had to be humanly euthanized and considered a fatality on day six post-viral inoculation. This further supports the idea that brain invasion is likely the main cause of lethality in this model.

The present study was aimed at evaluating the protective effect of ACE2 618-DDC-ABD in k18-hACE2 mice infected with the SARS-CoV-2 Delta variant to establish the universality of the in vivo protective effect of this ACE2 decoy protein. In previous in vitro studies, ACE2 618-DDC-ABD exerted a neutralizing effect on the infection of Vero E6 cells by the SARS-CoV-2 wild-type, Delta and Gamma variants at high concentrations [10,22,23]. Infection of A549 cells with the SARS-CoV-2 Omicron BA.1 variant was neutralized by 20-fold lower concentrations of ACE2-618-DDC-ABD than those required to neutralize the wild-type variant [23]. Some SARS-CoV-2 variants, like Omicron BA.1, may, therefore, be neutralized by markedly lower concentrations of ACE2-618-ABD-DDC.

The previous in vitro cell studies, together with the in vivo data in this study, show the universal protective effect of ACE2 618-DDC-ABD against SARS-CoV-2 variants. Mutational changes during viral evolution may lead to the development of resistance to current treatment approaches [24]. Strategies that have been employed to combat SARS-CoV-2 include monoclonal antibodies and specific antiviral drugs [58,59,60]. For those approaches applied in clinical practice, the development of mutations that compromise treatment efficacy has been described. In vitro studies have demonstrated the development of resistance toward the antiviral drugs Remdesivir and Paxlovid [61,62,63]. Moreover, the emergence of resistant mutations has been shown when in vitro passing the virus in the presence of SARS-CoV-2-specific monoclonal antibodies, highlighting the potential of rapidly occurring mutational changes [28,59,64]. Further studies have shown the development of resistant mutations in patients infected with the SARS-CoV-2 Delta and Omicron variants who received the monoclonal antibody Sotrovimab [65,66,67]. Moreover, mutations causing resistance against the antibody cocktails Casirivimab/Imdevimab (REGN-CoV-2) and Bamlanivimab/Etesevimab (LY-CoV555/LY-CoV016) have also been shown [68,69]. These drugs consist of two monoclonal antibodies targeting distinct epitopes [70,71,72], which is particularly interesting as combination therapies are typically less prone to mutational escape and decreased treatment efficiency [73,74,75,76]. The possibility of mutational escape has also been described for polyclonal antibodies, although, unlike monoclonal antibodies, a variety of different epitopes are being targeted [77,78].

In contrast to monoclonal antibodies, in vitro passaging of SARS-CoV-2 in the presence of a soluble ACE2 decoy protein did not lead to the development of resistant mutations [28]. Importantly, saturation mutagenesis of the SARS-CoV-2 RBD, followed by in vitro selection with full-length ACE2 and an engineered ACE2 decoy protein, did not show any RBD mutations that discriminated in favor of full-length ACE2 compared to the decoy protein [26]. Although the development of mutations that compromise the affinity for ACE2 decoy binding is theoretically possible, ACE2 decoy proteins clearly have less susceptibility to resistance caused by SARS-CoV-2 mutations [26,27]. Mutations that decrease decoy affinity would simultaneously decrease affinity for full-length membrane-bound ACE2, which is essential for the cell entry and infection of all currently known SARS-CoV-2 variants [8,9,10]. In fact, mutational escape from the neutralizing effect of ACE2 decoy proteins would, therefore, come at the expense of diminished viral infectivity and virulence [26,27].

During viral evolution, different mutations have been described that increase the affinity of the SARS-CoV-2 RBD for ACE2 receptor binding as, for example, the amino acid exchanges N501Y in the SARS-CoV-2 Alpha and Beta variants, as well as L452R in the SARS-CoV-2 Delta variant [48,79,80]. The current dominant circulating SARS-CoV-2 Omicron variants also harbor various spike protein mutations, leading to increased ACE2 receptor binding while efficiently evading neutralizing antibodies [81,82,83]. Consistent with this, as we have previously shown and noted above, the concentrations of ACE2 618-DDC-ABD required to neutralize the SARS-CoV-2 Omicron BA.1 variant in vitro are significantly lower than those needed to neutralize the wild-type variant [23]. Of note, while this might be explained by the higher binding affinity of the SARS-CoV-2 RBD to ACE2, in vitro studies with SARS-CoV-2 Omicron variants have also shown decreased fusion and syncytium formation, which may, in turn, result in lower concentrations being effective for the neutralization of the SARS-CoV-2 Omicron variant than those needed for other SARS-CoV-2 variants [83,84,85].

The present study also shows that kidney SARS-CoV-2 titers in k18-hACE2 mice infected with the SARS-CoV-2 Delta variant are undetectable. In our previous study with k18-hACE2 mice infected with the ancestral SARS-CoV-2 variant, kidney SARS-CoV-2 titers were also undetectable [22]. Of note, as the limit of detection for the plaque assay is 10^2^ PFU/mL, kidney viral titers might be either absent or below the limit of detection. While renal involvement has been recognized as a frequent complication of COVID-19, with an increased occurrence of acute kidney injury and associated higher mortality in hospitalized COVID-19 patients, the contributing mechanisms remain unclear [86,87,88,89]. Direct SARS-CoV-2 infection and viral replication in the kidney parenchyma have been discussed, as ACE2 is highly expressed in many cell types present in the kidney, like proximal tubular cells and podocytes [90,91,92]. SARS-CoV-2 has been shown to directly infect human kidney organoids, and the presence of SARS-CoV-2 in the kidney parenchyma of patients with COVID-19 has been demonstrated using methods like immunohistochemistry (IHC), immunofluorescence (IF), real-time PCR and single-cell RNA sequencing [93]. These data, however, are mainly derived from autopsies where the post-mortem interval from death to autopsy ranges from several hours to days, during which SARS-CoV-2 could spread to and within the kidney post-mortem [93]. Many studies have shown negative findings regarding the presence of SARS-CoV-2 in the kidney, including a large series of kidney biopsy samples (n = 284) of patients with COVID-19, where no direct kidney SARS-CoV-2 infection could be demonstrated [94]. Moreover, results from our group in the k18-hACE2 mouse model infected with the ancestral SARS-CoV-2 variant also showed a lack of evidence for kidney invasion [22,95]. The kidney tissue of k18hACE2 mice infected with the ancestral SARS-CoV-2 variant showed no detectable viral titers by plaque assay [22]. In addition to undetectable viral titers by plaque assay, there was no evidence for viral spike and nucleoprotein using IHC and IF staining [95]. Further analysis using single-molecule fluorescence in situ hybridization was also negative for SARS-CoV-2 RNA [95]. Undetectable kidney viral titers in k18-hACE2 mice infected with the Delta SARS-CoV-2 variant support the findings from our previous study. Considering the conflicting data throughout the literature, the question of whether direct kidney invasion by SARS-CoV-2 occurs remains not fully elucidated. Multiple factors, such as the timing between the confirmation of SARS-CoV-2 infection and obtaining the kidney sample, the type of sample obtained (biopsy or autopsy), as well as the sensitivity of the methods to detect the potential presence of SARS-CoV-2, may influence the detection of SARS-CoV-2 in the kidney [93,96].

## 5. Conclusions

The present study demonstrates the efficacy of ACE2 618-DDC-ABD to protect k18-hACE2 mice from lethal infection with the aggressive SARS-CoV-2 Delta variant. Animals that received ACE2 618-DDC-ABD before and after viral inoculation had significantly improved survival, mitigated weight loss and improved clinical scores. Moreover, lung and brain SARS-CoV-2 titers were significantly reduced in the ACE2-618-DDC-ABD-treated group. Importantly, brain viral titers were much higher than lung viral titers and appear much more likely to be the cause of lethality in the k18-hACE2 model. Taken together with previous in vivo experiments with wild-type SARS-CoV-2 and in vitro data on the neutralizing effect on various variants, including wild-type, Gamma, Delta, and Omicron BA.1 [22,23], this study shows the universality of the protective effect of ACE2 618-DDC-ABD against infection with different SARS-CoV-2 variants. Its therapeutic and preventive potential in humans should be investigated for current and future emerging coronaviruses that use ACE2 as their main receptor for cell entry.

## Data Availability

Data are contained within the article.
